# S15-3 24-hour movement behaviours and cognitive health of adults aged 55 years and over: cross-sectional findings of the PASOCA-study

**DOI:** 10.1093/eurpub/ckad133.074

**Published:** 2023-09-11

**Authors:** Pieter-Jan Marent, Genevieve Albouy, Greet Cardon, Jannique van Uffelen

**Affiliations:** KU Leuven, Belgium; Ghent University, Belgium; KU Leuven, Belgium; University of Utah, USA; Ghent University, Belgium; KU Leuven, Belgium

## Abstract

**Purpose:**

Increased longevity is one of the greatest success stories in public health. However, ageing is accompanied by cognitive decline which affects people’s daily functioning and, if it develops to dementia, their ability to live independently. The aim of this study is to precisely examine how the 24-hour movement behaviours, including physical activity (PA), sedentary behaviour (SB) and sleep, are related to cognitive function and which role these modifiable lifestyle factors can play in optimal cognitive ageing.

**Methods:**

The PASOCA-study is an observational, longitudinal study spanning over three years. Objective and subjective data of healthy, community dwelling adults aged 55 years and above are collected once a year. Participants wear an accelerometer (ActiGraph wGT3X-BT) on the wrist of their non-dominant hand for seven consecutive days and nights in order to objectively capture their 24-h movement behaviours. In addition, the International PA Questionnaire Short Form, a muscle-strengthening exercise (MSE) survey and the Pittsburgh Sleep Quality Index are administered to gather subjective input. Cognition (executive function and memory) is objectively tested with the Cambridge Neuropsychological Test Automated Battery (CANTAB) and subjectively with the Cognitive Failure Questionnaire (CFQ).

**Results:**

Baseline data collection took place from August 2021 till March 2022 and 234 participants (50.9% women; aged 68.3 ± 7.7 years) were measured. Multiple linear regressions and compositional data analyses will be conducted to understand the precise relationship between PA, SB, sleep and cognition (i.e. executive function and memory). The cross-sectional associations will be presented and discussed during the symposium.

**Conclusions:**

The resulting deeper understanding of the precise relationship between PA, SB, sleep and cognition will inform future guidelines and will contribute to the development of preventive interventions for maintaining cognitive health at older age.

**Support/Funding Source:**

Pieter-Jan Marent is funded by the Research Foundation Flanders (FWO) – 11B7123N.

